# Development of a Training Program for Epidemiological Investigation of Outbreaks of Unknown Cause

**DOI:** 10.56786/PHWR.2026.19.38.1

**Published:** 2026-09-08

**Authors:** Mina Ha, Eun-Hye Kim, Miryoung Yoon, Soyeon Kim, Jin Gwack, Ho-Jang Kwon, Won Kim, Inja Choi, Younjhin Ahn, Sunhye Choi, Seung-Hui So, Hae-Kwan Cheong, Bo Youl Choi

**Affiliations:** 1 Department of Preventive Medicine, Dankook University College of Medicine Cheonan Korea; 2 Department of Social and Preventive Medicine, Sungkyunkwan University School of Medicine Suwon Korea; 3 Department of Preventive Medicine, Jeonbuk National University Medical School Jeonju Korea; 4 Wonjin Institute for Occupational and Environmental Health Seoul Korea; 5 Division of Climate Change and Health Hazard, Department of Health Hazard Response, Korea Disease Control and Prevention Agency Cheongju Korea; 6 Department of Preventive Medicine, Hanyang University College of Medicine Seoul Korea

**Keywords:** Unexplained illness cluster, Epidemiological investigation, Training program, Non-infectious health risks, Interagency collaboration

## Abstract

**Objectives:**

Clusters of unexplained illness occurrence can span multiple administrative domains, delaying initial response and role coordination. Non-infectious clusters with unclear exposures, long latency periods, or low disease specificity are particularly difficult to investigate and manage. This study describes the development and pilot implementation of a training program for epidemiological investigation for such clusters.

**Methods:**

We synthesized materials from a 3-year project conducted during 2024–2026, including the initial proposal, expert reviews, presentation materials, reports, educational textbooks, pilot training records, and satisfaction survey results. The program was refined through research meetings, expert discussions, workshops, demonstrations, and the production of textbooks and video materials. Delivery of these materials was done in the form of online didactic sessions and scenario-based practical training that was conducted online and in person.

**Results:**

The program covered the concept and policy relevance of unexplained clusters, relevant investigation procedures, the generation of hypotheses, methods of data collection, risk communication strategies, techniques of interagency coordination, and case-based approaches to decision-making. Scenario exercises were designed to help trainees work through problem definition, exposure assessment, stakeholder coordination, interpretation of uncertain evidence, and communication of interim findings. The program also emphasized the policy blind spots that were created by unclarity of jurisdiction and the need to coordinate public health responses with environmental health functions.

**Conclusions:**

Training for non-infectious clusters of unexplained illness should go beyond lecture-based knowledge transfer and include problem-based learning, scenario discussions, and practical coordination exercises. Continuous revision based on field cases and formal evaluation of educational effects are needed for sustainable implementation.

**Key messages**

**① What is known previously?**

Although the Republic of Korea has well-established training and response systems for infectious disease investigations, non-infectious clusters of unknown causes or exposures and long latency periods can evade concentrated attention from authorities concerned with public health, environmental health, chemical safety, and disaster response, leaving investigative authority and collaboration unclear.

**② What new information is presented?**

From 2024 to 2026, basic and advanced courses, real-case scenario exercises, report-writing and evaluation guidelines, and a case archive were developed in stages to strengthen the capacity to address policy gaps.

**③ What are implications?**

For integration into regular epidemiological investigator training, a legal basis to clarify investigative authority, interagency procedures that would incorporate the participation of Korea Disease Control and Prevention Agency, objective tools of assessment, and a sustainable case archive system are needed.

## Introduction

Besides infectious diseases, health problems occurring in clusters may arise from a range of factors, including environmental pollution, chemical substances, climate-related disasters, and changes in the social and physical environment. Among these, noninfectious clusters where the cause or source of exposure cannot be immediately identified generate scientific and administrative uncertainty. During the initial stages of an investigation, it may be unclear whether the event represents an infectious or environmentally related disease, a chemical accident, an occupational exposure, or a health effect following a disaster. Consequently, ambiguity may arise regarding the agency that should lead the response and the legal procedures and investigation methods that should be applied. In particular, for diseases with very long latency periods between exposure to a specific hazardous agent and disease onset and with low disease specificity, identifying the cause at an early stage and establishing appropriate countermeasures can be extremely difficult. Such ambiguity and lack of specificity may create policy blind spots until the cause of the event is identified, potentially leading to delays in the initial investigation, avoidance of responsibility, insufficient data sharing, and delays in providing support to affected individuals.

The Republic of Korea (ROK)’s epidemiological investigation system for infectious diseases has been developed based on established legal authority and standardized procedures. By contrast, noninfectious or environmental health risk events may fall at the interface between the public health investigation capacity of the Korea Disease Control and Prevention Agency (KDCA) and the environmental health and chemical safety responsibilities of the Ministry of Climate, Energy and Environment. The Ministry of Climate, Energy and Environment is responsible for environmental health activities aimed at preventing and managing the health risks of environmental pollution, as well as the management of chemical substances and response to chemical accidents [1]. In addition, under the Framework Act on Health and Medical Services, the Ministry of Health and Welfare and the KDCA are required to establish measures to protect and promote public health, including maintaining a healthy environment, preventing health risks caused by environmental pollution, assessing the health effects of climate change, and preventing health risks resulting from climate change [2]. Therefore, noninfectious clusters of unexplained illness should be understood not as the sole responsibility of a particular ministry but as a collaborative issue requiring the integration, from the stage before cause identification, of public health investigation, environmental exposure assessment, chemical safety, disaster response, and field response by local governments.

However, until recently, training programs designed to improve epidemiological investigators’ and relevant personnel’s understanding of interagency collaboration and integrated, multidisciplinary approaches required in these situations and strengthen their capacity to respond rapidly when such events occur have been insufficient. Simply delivering knowledge of noncommunicable diseases is insufficient training to handle such events. Trainees must be able to establish a broad range of potential causes based on limited initial information; describe health effects by time, place, and person; assess environmental exposure and toxicological possibilities; coordinate investigation authority with related agencies; and draft investigation reports specifying uncertainties.

This study aimed to systematically describe the process and outcomes of an epidemiological investigation training program for noninfectious clusters of unexplained illnesses that was developed and pilot-tested in stages from 2024 to 2026. We sought to describe the policy needs motivating the program, the development procedures undertaken, and the educational curriculum through which these needs were translated into practice. In addition, based on the findings from pilot implementation, satisfaction surveys, and expert evaluations, we aimed to identify the requirements for future integration of the program into regular training curriculums.

## Methods

### 1. Education Program Development Model

In curriculum development, it is important to systematically organize needs assessment, goal setting, educational strategies, implementation, and evaluation [3]. The Analysis, Design, Development, Implementation, and Evaluation model is widely used in general instructional design and provides a useful framework for systematically structuring the development of an education program through five stages [4]. In this study, these educational principles were used as a reference for the program development and implementation process conducted by the research team. The process comprised needs assessment, formulation of educational goals and content, program development, and pilot implementation and evaluation, with the outputs of each stage incorporated into the subsequent stage. A cyclical process was incorporated such that the program could be revised based on findings identified through pilot implementation and evaluation (Figure 1).

### 2. Development and Implementation of the Education Program

In 2024, a research team comprising preventive medicine faculty members and field experts was formed, and the concept and scope of the target events were defined through research meetings, workshops, and meetings with the commissioning agency. The research team concluded that the conventional term “noninfectious diseases” did not adequately encompass public health events in which the cause and the responsibilities of individual ministries were unclear; therefore, it defined the target concept for the training as “health risk events of unknown etiology.” However, the program focused primarily on noninfectious diseases, for which epidemiological investigation experience is relatively limited compared with infectious diseases. Subsequently, based on educational needs, the research team established the educational purpose and learning objectives and developed and implemented theoretical instruction and scenario-based practical training. A satisfaction survey was conducted after completion of the training [4,5].

In 2025, areas requiring improvement and topics for advanced education were identified based on the results of the previous year’s satisfaction surveys, expert opinions, and discussions among the research team. The educational content and implementation methods were refined through regular meetings, discussions, and workshops, and theoretical instruction and scenario-based practical training were conducted in collaboration with the relevant department of the KDCA. After training completion, a satisfaction survey was conducted to evaluate the appropriateness of the curriculum and identify areas requiring improvement. In parallel, environmental health–related health risk events that had occurred in the ROK were reviewed, and a standardized procedure was established for developing a case archive through systematic collection and organization of case information [6].

In 2026, the advancement of the educational content and practical training system was promoted based on experience gained from implementing the 2024–2025 curriculum and the case archive. Through regular research team meetings, literature reviews, expert consultations, and workshops, new practical training scenarios, a results-report writing and evaluation system, and approaches for fostering practical training facilitators and expert instructors have been developed and refined. Operational procedures were also designed such that trainees would prepare a results report after practical training and receive feedback based on standardized evaluation criteria.

While drafting this manuscript, ChatGPT 5.6 Sol Light (OpenAI) was used as an auxiliary tool to improve sentence clarity and readability. The authors reviewed the final content and assume responsibility for it.

## Results

### 1. Policy Characteristics of Noninfectious Clusters of Unknown Cause

Noninfectious clusters of unexplained illness are characterized by unclear administrative jurisdiction until the cause of the disease is identified. When clustered health effects occur even after an infectious disease has been excluded or remains uncertain, rapid epidemiological investigation from a public health perspective is necessary. At the same time, such events may also be related to environmental health functions involving environmental pollution, chemical substances, indoor air quality, disaster-related exposures, and industrial or community environments. Therefore, the education program focused on the core competencies required to generate a broad range of potential causes, coordinating the roles of related organizations, and drafting investigation plans and reports while acknowledging uncertainty (Table 1).

In the expert evaluation of the 2026 proposal, key issues included a conceptual distinction from existing environmental health investigations, differences in investigative approaches, the participation of actual target trainees, and clarification of the unknown-cause component in the wildfire scenario. These findings indicate that the education program should develop competencies relevant to the interface between public health investigations and environmental health investigations during the period before the cause is identified.

### 2. Structure of the Education Program

In 2024, the research team established the basic concepts and terminology of the training program and developed a six-chapter basic course totaling 6 hours. The basic course comprised health risk events of unknown etiology, case studies of clusters, factors associated with their occurrence, methods for detecting clusters, methods for epidemiological investigation, and environmental exposure assessment. Each chapter included the educational purpose, learning objectives, and detailed learning content, and lecture materials and videos were produced for online instruction. Practical training and case exercises included an “epidemiological investigation of a high-exposure mercury cluster in inland Gyeongbuk” and an “epidemiological investigation of a cluster of severe lung disease of unknown etiology associated with humidifier disinfectants.” Each case was developed as a scenario with discussion tasks to ensure that trainees could progressively review limited information and formulate an investigation plan [5].

In 2025, based on feedback from the basic course, a five-chapter advanced course totaling 5 hours and additional practical training scenarios were developed. The advanced course covered the toxicity and health effects of hazardous agents, methods for exposure assessment involving hazardous agents and contaminated media, epidemiological investigation of climate-related disasters, and the detection of and preparedness for noninfectious health risks that could cause clusters in the near future. Lecture materials and videos were also produced to enable online delivery of the advanced course. For practical training and case exercises, the “epidemiological investigation of a pneumonia cluster at the College of Veterinary Medicine” and the “epidemiological investigation of an infant cyanosis event involving methemoglobinemia” were reconstructed to represent situations in which only limited information was available before cause identification. The exercises were designed such that trainees could progressively experience initial information analysis, case definition, establishment of a surveillance system, descriptive epidemiological analysis, environmental and toxicological investigation, conclusion development, and reporting [6].

In 2026, further development of the practical training scenarios and expansion of the training system were pursued concurrently. “Epidemiological investigation of the cancer cluster in Jangjeom village, Iksan” and the “Health impact survey of residents affected by a wildfire” were newly developed and added to the practical training scenarios. The Jangjeom village scenario focused on examining various environmental exposure factors and accumulating epidemiological evidence in a situation in which the initial cause was unclear. The wildfire health impact scenario was developed to strengthen the ability to assess evolving multiple hazards, evaluate health impacts and causality using limited data, communicate uncertainty, and link findings to long-term surveillance. In addition, the results-report template was refined to allow trainees to systematically organize the findings of practical training, and a “standard evaluation guide” was developed to assess the report’s structure and the appropriateness of the investigation process. Plans for fostering practical training facilitators and expert instructors and for operating workshops on preparedness for future clusters are also being developed as outputs intended to support expansion of the education system.

The overall structure of the education program that was actually developed and implemented is presented in Table 2.

### 3. Training Implementation and Evaluation Results

In 2024, theoretical instruction was delivered live online from November 27 to 28, and scenario-based practical training was conducted online on December 3 for 2.5 hours, with participants divided into two small groups for each case. A total of 38 participants attended the theoretical instruction, and 23 epidemiological investigators and related personnel participated in the practical training. A total of 20 participants responded to the satisfaction survey. Open-ended responses indicated that participants found the new topics and exercises based on actual events useful. Meanwhile, suggestions for improvement included adding lectures on toxic substances, providing in-person training, sharing simulation materials in advance, and extending the time allocated to practical training [5].

In 2025, theoretical instruction was delivered live online over 3 days beginning on November 11 and included the basic course developed in the previous year and the newly developed advanced course. Practical training was changed to an in-person format. A total of 57 participants attended the theoretical instruction (basic and advanced), and 25 epidemiological investigators and related personnel participated in the in-person practical training. A total of 12 participants responded to the satisfaction survey. The trainees reported that the scenarios were specific and structured in a stepwise manner and that the expert feedback on each group’s responses was useful. They also suggested that, because the available training time was insufficient, the theoretical instruction and scenario exercises should be divided between the first and second halves of the year [6].

The 2024 and 2025 projects received the highest rating in expert evaluations, with positive evaluations of the potential policy utility of the curriculum and case archive.

In 2026, two rounds of in-person training were planned. The first training session, conducted during the first half of the year, was held on June 15 as a 1-day program using existing practical cases. The theoretical instruction was attended by 14 participants, and the practical training included 13 epidemiological investigators and related personnel, of whom 12 responded to the satisfaction survey. Over 88% of the respondents evaluated the training positively, but all respondents indicated a need for additional training. The open-ended responses mentioned the utility of report-writing exercises based on the scenarios and presented clarifying the legal basis and training objectives and adding various cases as needs for improvement. After the practical training, trainees prepared results reports, which were evaluated by experts using the “standard evaluation guide,” and individualized feedback was provided to strengthen investigation and reporting competencies.

### 4. Development of the Case Archive and a Foundation for Educational Feedback

In 2025, a foundation was established for developing a case archive to support the continued use of case-based education. The research team reviewed 53 environmental health-related health risk events that had occurred in the ROK since industrialization and collected 63 relevant academic articles and 22 reports. Data-entry items were divided into two forms: the first for recording the epidemiological characteristics of health risk events of unknown etiology and the second for entering bibliographic information on articles and reports related to each event. Data entry was completed for 14 health risk events of unknown etiology, thereby providing examples of case standardization (Table 3) [6].

In 2026, a direction was proposed for using the case archive not only as a list of materials but also as a foundation for an educational feedback loop. Additional fields were incorporated into the case archive, including limitations in the epidemiological investigation process and findings; factors contributing to uncertainty, policy and institutional implications; and procedures for expert validation. The case archive may thereby serve as a foundation linking scenario development, trainee report writing, facilitator feedback, and improvement of the regular curriculum.

## Discussion

This project is significant in that it reframed noninfectious clusters of unexplained illness not as a new educational topic but as an issue of public health capacity required to address policy blind spots. Clusters occurring before cause identification may not be immediately assignable to any single administrative category, such as infectious disease control, environmental health, chemical safety, or disaster response. In such circumstances, epidemiological investigators require specialized knowledge of particular causes as well as the ability to consider multiple potential causes in uncertain situations, establish investigation priorities, coordinate data and roles among relevant agencies, and report findings while clearly indicating the level of evidence and associated uncertainty.

This perspective is particularly important at the interface between the environmental health functions of the Ministry of Climate, Energy and Environment and the public health investigation functions of the KDCA. Environmental health functions are concerned with preventing and managing health effects caused by environmental pollution and chemical substances, whereas clusters of unexplained illness require assessment of the magnitude and patterns of health effects even before the cause is identified. Therefore, this education program should not be positioned as an effort to replace environmental health investigations or assert jurisdiction but as common competency training intended to reduce gaps before the determination of jurisdiction and facilitate interagency collaboration.

From the perspective of program development, a key output was the progressive accumulation of actual courses and practical training scenarios. In 2024, online theoretical instruction focused on basic concepts and epidemiological investigation methods, along with online simulation exercises. In 2025, advanced topics with greater practical applicability, including environmental toxicology, exposure assessment, and climate-related disasters, were incorporated. The practical training format also evolved from remote simulation exercises to in-person scenario-based training. In 2026, results-report writing and an evaluation guide were added, moving the program beyond participatory exercises toward a structure in which investigation and reporting competencies could be assessed and incorporated into feedback.

Case-based scenario exercises are a particularly important strategy in this education program. In training for noninfectious clusters of unexplained illness, it is more important for trainees to encounter incomplete information and conflicting clues from the period before cause identification than to simply be informed of the conclusions of cases that have already been resolved. Trainees must formulate hypotheses based on limited information, determine what additional data are required, integrate exposure assessment with epidemiological data, and consider interagency collaboration and risk communication. Report writing and feedback after practical training provide opportunities to document and improve these decision-making processes.

A limitation of the current evidence is that it is based primarily on satisfaction surveys and expert evaluations. Satisfaction, perceived understanding, and self-reported improvement in competency can demonstrate the acceptability of the training and changes in perceptions, but they do not directly establish improvement in actual investigation competency, changes in field behavior, or enhanced response capacity at the institutional level. The writing of result reports and the evaluation guide introduced in 2026 represent an important transition toward behavioral-level evaluation. Future evaluation should incorporate objective assessment tools covering scenario report performance, pre- and post-training knowledge, the ability to develop investigation plans and describe uncertainty, and collaborative communication.

Several limitations should be considered in the development of this education program. First, the small number of respondents to the satisfaction surveys and the limited number of training participants make it difficult to generalize the outcomes of the development process. In addition, evaluation consisted of trainee satisfaction and expert evaluation of the projects; there is no objective evidence demonstrating changes in knowledge before and after training or improvement in actual investigation competency. Furthermore, clarification of investigative authority through the establishment of a legal basis for health risk events of unknown etiology, allocation of roles among ministries, and the scope of the target training population have yet to be sufficiently institutionalized. Consequently, uncertainty regarding these issues remains within the educational content itself.

In the future, a standardized curriculum for integration into regular training, a system for training instructors and facilitators, objective competency assessment tools, and a sustainable case archive system should be established simultaneously. In particular, interagency collaboration exercises should be explicitly incorporated into the curriculum. For example, training scenarios could specify what information is held by the KDCA, the Ministry of Climate, Energy and Environment, local governments, medical institutions, environmental monitoring organizations, and research institutions, as well as the respective authorities and limitations of these organizations. Trainees could then be asked to design the lead investigative structure, data-request procedures, field investigation activities, risk communication, and reporting systems. This is a key element toward expanding the education program from epidemiological skills to policy coordination competency.

In conclusion, the 2024–2026 epidemiological investigation training program for noninfectious clusters of unexplained illness represents a staged approach to curriculum development aimed at strengthening the capacity to respond to events that may fall within policy blind spots. This model may be used to strengthen national public-health response capacity for clusters of health risks of unknown etiology and establish a foundation for collaboration between public and environmental health.

## Figures and Tables

**Figure 1:**
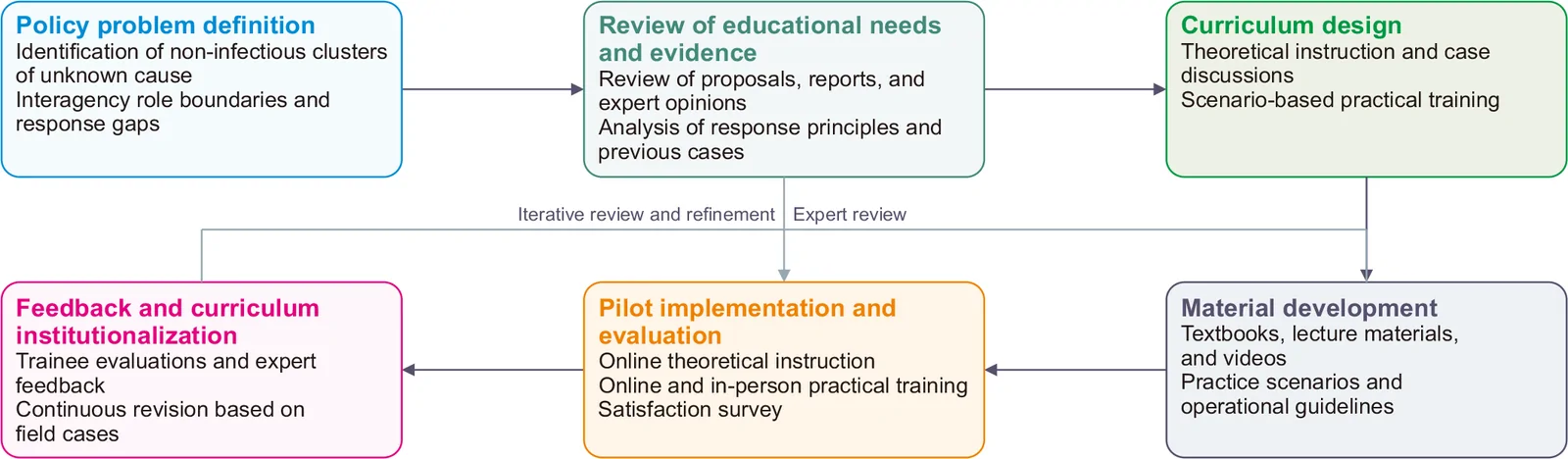
Development and feedback framework for the education program This figure represents that process of the development of an education program covering administrative and scientific uncertainty before the identification of a cause with research meetings and workshops, development of educational materials, program implementation and evaluation, feedback, and the institutionalization of the regular curriculum.

**Table 1:** Characteristics of policy blind spots and the educational competencies required in response

Policy characteristic	Field issue	Required educational competency	Training component
Unclear jurisdiction before the cause is identified	Uncertainty about which procedures should be prioritized among those of infectious disease control, environmental health, chemical safety, and disaster response	Initial situational assessment; development of potential causes; coordination of the lead investigative body	Concepts concerning health risk events of unknown etiology; training in detection and initial response
Overlapping interagency responsibilities	Concurrent investigations by public health authorities, environmental health authorities, and local governments are needed	Interagency communication; data sharing; allocation of roles	Collaborative scenarios; practice in developing an investigation plan
Scientific uncertainty	Exposure source, mechanism of occurrence, and causal relationship with health effects cannot be immediately confirmed	Hypothesis generation; assessment of environmental exposure; toxicological judgment; description of uncertainty	Environmental exposure assessment and environmental toxicology theory; case-based exercises
Need to translate investigation findings into policy	Investigation findings need to lead to concrete approaches to victim support, environmental management, and institutional improvement	Report writing; expression of levels of evidence; derivation of policy implications	Use of results-report template; evaluation guide; feedback

**Table 2:** Structure of the education program for the development and implementation of epidemiological investigation for outbreaks of unknown cause

	Theoretical instruction (1 hour each)a)	Scenario-based simulation training (2–4 hours each)b)
Basic	- Understanding health risk events (of unknown etiology) - Case studies of methods for detecting clusters of health risk events - Factors associated with the occurrence of health risk events - Methods of detecting clusters - Methods for the epidemiological investigation of health risk events - Understanding the assessment of environmental exposure in cases of epidemiological investigation	- Cluster of severe lung disease of unknown etiology associated with humidifier disinfectants - Epidemiological investigation of a high-exposure mercury cluster in inland Gyeongbuk
Advanced	- Understanding the toxicity and health effects of hazardous agents - Exposure assessment for hazardous agents and contaminated media: Part 1 - Exposure assessment for hazardous agents and contaminated media: Part 2 - Epidemiological investigation of climate-related disasters - Detection of and preparedness for health risks that may cause future clusters	- Outbreak of pneumonia at the College of Veterinary Medicine - Case of methemoglobinemia - Cancer cluster in Jangjeom village, Iksan - Health impact survey of residents affected by a wildfire

^a)^Online instruction using textbooks and videos; satisfaction assessment. ^b)^Writing a results report and feedback after scenario training; satisfaction assessment.

**Table 3:** Variables to characterize the epidemiological features of health risk events of unknown etiology in case archives (7 domains, 82 variables)

Major category	Subcategory	Variables
1. Basic event information	Event identification	Event ID (unique number assigned according to the classification system); event name (Korean/English)
	Spatial information	Location of occurrence (province/city–county–district/township–neighborhood); type of setting (residence, workplace, school, medical institution, etc.); geographic extent (radius/area)
	Temporal information	Date of onset of the first symptom (estimated); date of recognition of cluster; date and time of the initial report or notification; date and time of the first media report; date that the official investigation began; date that the cause was announced
	Initial recognition pathway	First recognizer (resident, medical institution, media, public official, researcher, etc.); first reporting institution; pathway of social recognition (media organization, civic group, and government announcement, etc.)
2. Epidemiological characteristics	Magnitude of health impact	Type of health outcome (disease/syndrome); affected population (total exposed population); number of suspected cases; number of confirmed cases; number of deaths; number of hospitalizations
	Epidemiological indicators	Incidence; prevalence; case-fatality rate; excess mortality (if applicable)
	Demographic characteristics	Sex distribution; age-group distribution; occupational distribution; characteristics of high-risk groups
	Clinical characteristics	Major symptoms and signs; latency or induction period until onset of symptoms; distinctive features of the clinical course
	Temporal distribution	Shape of the epidemic curve; presence of seasonality or periodicity
	Spatial distribution	Geographic concentration; presence of spatial clusters
3. Exposure characteristics	Contaminant	Suspected causal agent; confirmed causal agent; methods used to identify the cause; characteristics of the causal agent (namely, chemical, physical, biological, etc. characteristics)
	Source	Type of source (factory, mine, landfill, water supply, etc.); location of source; beginning of operation of source; number/scale of sources
	Pollution provider	Primary responsible company/organization; whether legal liability was acknowledged
	Exposure pathway	Main route of exposure (inhalation, oral ingestion, dermal contact, etc.); medium of exposure (air, water, soil, food, etc.); environment of exposure (indoor/outdoor; occupational/environmental)
	Environmental monitoring	Date and time when environmental samples were collected; analytes; whether environmental standards were exceeded
4. Investigation and research	Epidemiological investigation	Institution performing the epidemiological investigation; start date of the investigation; period of the investigation; methods; number of participants; major findings
5. Response and measures	Initial response	First responding institution/organization; date and time that response began
	Response before cause identification	Whether an environmental investigation was conducted; whether medical support was provided; whether source operations were suspended; other preventive measures taken
	Response after cause identification	Whether the source was removed or blocked; whether health examinations were provided to residents; whether environmental remediation was undertaken
	Legal/administrative measures	Administrative sanctions; amount and payment status of compensation
	Long-term management	Whether follow-up observation was conducted; whether a post-event monitoring system was established
6. Socioeconomic impact	Scale of damage	Direct economic losses; indirect economic losses; number of displaced residents
7. Discussion and implications	Institutional change	Whether relevant laws were enacted or amended; whether environmental health standards changed; whether a policy was introduced or revised
	Investigation limitations and uncertainty	Limitations in the epidemiological investigation process; factors contributing to uncertainty in interpretation; unresolved issues and need for further investigation
	Policy and institutional implications	Institutional gaps or deficiencies; implications of policies; recommendations for the prevention of recurrence
