# Supplementary material for: Development of a Training Program for Epidemiological Investigation of Outbreaks of Unknown Cause
Source: Public Health Wkly Rep. 2026 Sep 8;19(38):1599–619. doi: 10.56786/PHWR.2026.19.38.1 (PMC13654660; doi:10.56786/PHWR.2026.19.38.1)
Supplement: Translation

# Translation: 원인미상 집단발병 역학조사를 위한 교육프로그램 개발

**Authors:** 미나 하, 은혜 김, 미령 윤, 소연 김, 진 곽, 호장 권, 원 김, 인자 최, 윤진 안, 선혜 최, 승휘 소, 해관 정, 보율 최

**Affiliations:** 1 단국대학교 의과대학 예방의학교실; 2 성균관대학교 의과대학 사회의학교실; 3 전북대학교 의과대학 예방의학교실; 4 원진재단부설 노동환경건강연구소; 5 질병관리청 건강위해대응관 기후보건ㆍ건강위해대비과; 6 한양대학교 의과대학 예방의학교실

**Keywords:** 원인미상 집단발병, 역학조사, 교육프로그램, 비감염성 건강위해, 다부처 협업

## Abstract

**목적:**

원인미상 집단발병은 감염병, 환경보건, 산업보건, 식품안전 등 여러 행정 영역에 걸쳐 발생할 수 있어 초기 대응과 역할 조정이 지연될 위험이 있다. 특히 원인이나 노출원이 불분명하고 증상의 발현이 노출로부터 장시간이 소요되거나 질환의 특이성이 낮은 비감염성 집단발병은 원인 규명과 대책 수립이 어렵다. 이 연구는 원인미상 비감염성 집단발병에 대한 역학조사 역량을 높이기 위한 교육프로그램을 개발하고 시범운영한 과정을 제시하였다.

**방법:**

2024–2026년 수행한 정책연구용역을 중심으로 교육 수요, 국내외 대응 원칙, 전문가 검토 의견, 연구진 회의와 워크숍 결과를 종합하였다. 이를 토대로 이론 강의, 사례 기반 실습 시나리오, 교재와 동영상 자료를 개발하고, 비대면 이론교육과 비대면 및 대면 실습교육을 운영한 뒤 만족도 조사를 실시하였다.

**결과:**

교육프로그램은 원인미상 비감염성 집단발병의 개념과 정책적 쟁점, 역학조사 절차, 위험소통, 다부처 협업, 사례 분석, 현장 의사결정 실습을 포함하도록 구성되었다. 특히 조사 초기의 불확실성, 부처 간 업무영역 중첩, 기후ㆍ환경 요인과 보건 영향의 연결 가능성을 다루도록 설계하였다. 시범운영에서는 강의와 시나리오 토론을 결합하여 교육생이 문제 정의, 가설 설정, 자료 수집, 관계 기관 협의, 결과 해석을 단계적으로 수행하도록 하였다.

**결론:**

원인미상 비감염성 집단발병 대응 역량은 단일 분야 지식보다 문제 기반 학습, 사례 토론, 협업 절차 훈련을 통해 강화될 수 있다. 향후 정규 교육 과정으로 확산하기 위해 교육 효과 평가와 실제 대응 사례에 기반한 지속적 개선이 필요하다.

**핵심요약**

**① 이전에 알려진 내용은?**

국내 감염병 역학조사 교육과 대응체계는 비교적 체계화되어 있으나, 원인 또는 노출원이 즉시 확인되지 않고 증상의 발현이 노출로부터 장시간이 소요되는 비감염성 집단발병은 보건, 환경보건, 화학안전, 재난대응 등의 경계에 놓여 조사 주체와 협업 방식이 불명확해질 수 있다.

**② 새로이 알게 된 내용은?**

2024–2026년 과제를 통해 기본 이론교육, 심화 이론교육, 실제 사례 기반 시나리오 실습, 보고서 작성과 평가가이드, 사례 아카이브가 단계적으로 개발되었다. 이 프로그램은 정책 사각지대 대응 역량을 함양하기 위한 목적을 가진다.

**③ 시사점은?**

역학조사관의 정규 교육으로 확장하려면 원인미상 비감염성 집단발병의 법적 근거 마련을 통한 조사 권한 명확화, 질병관리청과 관계 부처의 협업 절차, 객관적 실습 평가도구, 사례 아카이브 운영체계가 함께 마련되어야 한다.

## 서 론

집단적으로 발생하는 건강 문제는 감염병뿐 아니라 환경오염, 화학물질, 기후재난, 사회ㆍ물리적 환경 변화 등 다양한 요인에 의해 발생할 수 있다. 이 중 원인 또는 노출원이 즉시 확인되지 않는 비감염성 집단발병은 과학적 불확실성과 행정적 불확실성을 동시에 낳는다. 조사 초기에는 감염병인지, 환경성 질환인지, 화학사고인지, 직업성 노출인지, 재난 이후 건강영향인지가 확정되지 않기 때문에 어느 기관이 주관하고 어떤 법적 절차와 조사 방법을 적용할지 모호해질 수 있다. 특히 특정 위해요인에 노출된 후 질병 발생까지 지연기간이 매우 길고 질병의 특이성이 크지 않은 질환의 경우 그 원인을 조기에 찾고 대책을 수립하는 것이 매우 어려울 수 있다. 이러한 모호성과 비특이성은 사건의 원인이 규명될 때까지 정책적 사각지대를 만들고, 초기 조사 지연, 책임 회피, 자료 공유 부족, 피해자 지원 지연으로 이어질 위험이 있다.

국내 감염병 역학조사 체계는 법적 근거와 표준 절차를 바탕으로 발전해 왔다. 반면 비감염성 또는 환경성 건강위해사건은 질병관리청의 공중보건 조사 역량과 기후에너지환경부의 환경보건ㆍ화학안전 업무가 만나는 경계에 놓일 수 있다. 기후에너지환경부는 환경오염이 건강에 미치는 영향을 예방ㆍ관리하기 위한 환경보건 업무와 화학물질ㆍ화학사고 대응 업무를 수행하고 있다[1]. 또 「보건의료기본법」에 따라 보건복지부와 질병관리청은 쾌적한 환경의 유지와 환경오염으로 인한 건강상의 위해 방지, 기후변화에 따른 국민보건영향 평가 및 기후변화로 인한 건강상의 위해 방지 등 국민의 건강을 보호증진하기 위한 시책을 강구하여야 한다[2]. 따라서 원인미상 비감염성 집단발병은 특정 부처의 단독 업무라기보다, 원인 규명 전 단계부터 공중보건 조사, 환경노출평가, 화학안전, 재난대응, 지방자치단체 현장 대응이 결합되어야 하는 협업 문제로 이해할 필요가 있다.

그러나 최근까지 역학조사관과 관련 업무 담당자들이 이러한 여러 부처의 협업과 다양한 영역의 결합적 접근이 필요한 부분에 대한 이해를 높이고 사건 발생 시 빠르게 대처할 수 있는 역량을 강화하기 위한 교육프로그램이 충분하지 않았다. 이러한 사건을 다루는 교육은 단순히 비감염성 질환 지식을 전달하는 방식으로는 충분하지 않다. 교육생은 제한된 초기 정보를 바탕으로 원인 후보를 넓게 설정하고 시간ㆍ장소ㆍ사람별 건강영향을 기술하며, 환경노출과 독성학적 가능성을 평가하고 관련 기관과 조사 권한을 조정하며, 불확실성을 명시한 조사보고서를 작성할 수 있어야 한다.

본 연구의 목적은 2024년부터 2026년까지 단계적으로 개발 및 시범운영된 원인미상 비감염성 집단발병 역학조사 교육프로그램의 과정과 결과를 체계적으로 기술하는 것이다. 이 프로그램이 어떤 정책적 필요에서 출발했고 어떤 절차를 거쳐 어떤 교육과정으로 구현되었는지 기술하고자 한다. 또한 시범운영과 만족도 조사, 전문가 평가 결과를 바탕으로 향후 정규 교육과정화를 위한 과제를 제시하고자 한다.

## 방 법

### 1. 교육프로그램 개발 모형

교육과정 개발에서는 요구분석, 목표 설정, 교육전략, 실행, 평가를 체계화하는 접근이 중요하며[3], 일반 교수설계에서 활용되는 Analysis, Design, Development, Implementation, Evaluation 모형은 분석, 설계, 개발, 실행 및 평가의 다섯 단계에 따라 교육프로그램의 개발 절차를 체계적으로 구성하는 데 참고할 수 있다[4]. 본 연구에서는 이러한 교육이론을 실제 연구진의 개발ㆍ운영 과정에서 참고틀로 활용하였다. 즉 요구분석부터 교육목표 및 내용 설계, 프로그램 개발과 시범운영, 평가에 이르는 단계로 구성하였으며, 각 단계의 산출물이 다음 단계에 반영되도록 하였다. 또한 시범운영과 평가에서 확인된 사항을 바탕으로 프로그램을 보완할 수 있도록 순환적 과정을 포함하였다(그림 1).

### 2. 교육프로그램 개발과 운영

2024년에는 예방의학 교수진과 현장 전문가로 연구진을 구성하고, 연구회의, 워크숍 및 발주기관과의 간담회를 통해 교육 대상의 개념과 범위를 설정하였다. 연구진은 기존 비감염성질환이라는 표현이 원인과 부처별 책임 소재가 불분명한 공중보건 사건을 충분히 포괄하지 못한다고 보고, 교육 대상 개념을 원인미상 건강위해사건으로 정리하였다. 다만 감염성 질환에 비해 역학조사 경험이 상대적으로 부족한 비감염성 질환에 중점을 두었다. 이후 교육 요구를 바탕으로 교육 목적과 학습목표를 설정하고 이론교육과 시나리오 기반 실습훈련을 개발ㆍ운영하였으며, 교육 종료 후 만족도 조사를 실시하였다[4,5].

2025년에는 전년도 만족도 조사 결과, 전문가 의견 및 연구진 논의를 바탕으로 개선 요구와 심화교육 항목을 도출하였다. 정기적인 회의와 토론, 워크숍을 통해 교육 내용과 운영 방식을 보완하고 질병관리청 담당부서와 협력하여 이론교육과 시나리오 기반 실습훈련을 운영하였다. 교육 종료 후 만족도 조사를 실시하여 교육 과정의 적절성과 개선 필요 사항을 평가하였다. 이와 함께 국내 환경보건 관련 건강위해사건을 검토하고 사례 정보를 체계적으로 수집ㆍ정리하기 위한 표준화된 아카이브 구축 절차를 마련하였다[6].

2026년에는 2024–2025년 교육 과정의 운영 경험과 사례 아카이브를 바탕으로 교육 내용과 실습체계의 고도화를 추진하였다. 정기 연구진 회의, 문헌조사, 전문가 자문 및 워크숍을 통해 신규 실습 시나리오, 결과보고서 작성 및 평가 체계, 실습 퍼실리테이터와 전문 강사진 양성 방안을 개발ㆍ보완하고 있으며 실습 후 교육생이 작성한 결과보고서에 표준화된 평가 기준에 따라 피드백을 제공하도록 운영 절차를 설계하였다.

본 연구의 원고 작성 과정에서 문장의 명료성과 가독성을 개선하기 위해 ChatGPT 5.6 Sol Light (OpenAI)를 보조적으로 활용하였으며 최종 내용은 저자가 검토하고 책임졌다.

## 결 과

### 1. 원인미상 비감염성 집단발병의 정책적 특징

원인미상 비감염성 집단발병은 질병의 원인이 규명되기 전까지 행정적 소관이 불명확한 특징을 보인다. 감염병이 배제되거나 명확하지 않은 상태에서도 집단적 건강피해가 발생하면 공중보건 관점의 신속한 역학조사가 필요하지만, 동시에 환경오염, 화학물질, 실내공기질, 재난 노출, 산업ㆍ지역 환경 등 환경보건 업무와도 연결될 수 있다. 이 때문에 교육프로그램은 특정 질환에 대한 지식보다 원인 후보를 넓게 설정하고, 관련 기관의 역할을 조정하며, 불확실성을 드러낸 상태에서 조사계획과 보고서를 작성하는 역량을 핵심으로 삼았다(표 1).

2026년 제안서 전문가 평가에서도 기존 환경보건 조사와의 개념적 차이, 접근 방법의 차이, 실제 교육 수요자 참여, 산불 시나리오에서 원인미상 요소의 명확화가 주요 쟁점으로 제시되었다. 이는 이 교육프로그램이 원인 규명 이전 단계에서 공중보건 조사와 환경보건 조사가 만나는 접점의 역량을 훈련해야 함을 보여준다.

### 2. 개발된 교육프로그램의 구성

연구진은 2024년에 교육의 기본 개념과 용어를 정립하고 6개 장, 총 6시간의 기본 교육 과정을 개발하였다. 기초 교육은 원인미상 건강위해사건의 이해, 집단발병 사례, 발생 관련 요인, 감지 방법, 역학조사 방법, 환경노출평가의 이해로 구성되었다. 각 장에는 교육 목적, 학습목표 및 세부 학습내용을 제시하였으며, 온라인 교육에 활용할 수 있는 강의자료와 강의 동영상을 제작하였다. 실전훈련 및 사례로는 ‘경북 내륙지역 수은 고노출 집락 역학조사’와 ‘원인미상 중증 폐질환 사건(가습기살균제) 역학조사’로 구성하였다. 각 사례는 교육생이 제한된 정보를 단계적으로 검토하고 조사계획을 수립할 수 있도록 시나리오와 토론 과제로 제작하였다[5].

2025년에는 기본 교육의 피드백을 바탕으로 5개 장, 총 5시간의 심화 교육과 실습 교육이 보완되었다. 심화 교육에는 유해인자의 독성과 건강영향, 유해인자와 매체 오염 등 노출평가 방법, 기후재난 역학조사, 가까운 미래에 집단발병이 가능한 비감염성 건강위해 발굴과 대비가 포함되었다. 심화 교육도 온라인 활용이 가능하도록 강의자료와 동영상으로 제작하였다. 실전훈련 및 사례로는 ‘A대 동물생명과학대학 폐렴 집단발병 역학조사’와 ‘영아 청색증 발생 사건(메트헤모글로빈혈증) 역학조사’ 사례를 원인 규명 이전의 제한된 정보 상황으로 재구성하여, 교육생이 초기 정보 분석, 환례 정의, 감시체계 구축, 기술역학 분석, 환경ㆍ독성 조사, 결론 도출과 보고 과정을 단계적으로 경험하도록 설계하였다[6].

2026년에는 실습사례집 고도화와 교육체계 확장이 함께 추진되었다. ‘익산 장점마을 집단 암발병 사건 역학조사’와 ‘산불지역 주민 건강영향 조사’가 신규로 개발되어 실습 시나리오에 추가되었다. 익산 장점마을 시나리오는 초기 원인이 명확하지 않은 상태에서 다양한 환경노출 요인을 검토하고 역학적 근거를 축적하는 과정에 초점을 두었다. 산불 발생 지역 주민 건강영향 조사 시나리오는 시공간적으로 변화하는 복합 위해요인을 분석하고, 제한된 자료로 건강피해와 인과성을 판단하여 불확실성과 함께 소통하고 중장기 감시로 연계하는 역량을 기르도록 개발되었다. 또한 교육생이 실습 결과를 체계적으로 정리할 수 있도록 결과보고서 작성 양식을 보완하고, 보고서의 구성과 조사 과정의 적절성을 평가하기 위한 ‘표준 평가 가이드’를 개발하였다. 이와 함께 실습 퍼실리테이터와 전문 강사진 양성 방안 및 미래 집단발병 대비 워크숍 운영안을 교육체계 확장을 위한 산출물로 마련하고 있다.

실제 개발ㆍ운영된 교육프로그램의 전체 구성은 표 2와 같다.

### 3. 교육운영 및 평가 결과

2024년 이론교육은 11월 27일부터 28일까지 실시간 온라인 방식으로 운영하였고, 시나리오 기반 실습훈련은 12월 3일 사례별 2개 소그룹으로 나누어 온라인에서 2시간 30분 동안 진행하였다. 이론교육에는 38명이, 실습훈련은 23명의 역학조사관 등이 참여하였고, 만족도 조사에는 20명이 응답하였다. 자유 응답의 내용을 통해 새로운 주제와 실제 사건 기반 실습의 유익성이 확인되었다. 동시에 독성물질 강의 추가, 대면교육 필요, 모의훈련 자료 사전 공유, 실습 시간 확대 등 개선 요구도 제기되었다[5].

2025년 이론교육은 11월 11일부터 3일간 전년도에 개발한 기초교육과 신규 심화교육을 함께 제공하는 실시간 온라인 방식으로 운영하였으며, 실습훈련은 대면 방식으로 전환하였다. 이론교육(기초 및 심화)에는 57명이, 대면으로 실시된 실습훈련에는 25명의 역학조사관 등이 참여하였다. 만족도 조사는 12명이 응답하였다. 교육생 의견에서는 시나리오가 구체적이고 단계별로 구성되어 있으며 조별 답변에 대한 전문가 피드백이 유익했다는 평가가 있었고, 교육 시간이 부족하므로 이론교육과 시나리오를 상ㆍ하반기로 나누어 운영하자는 제안도 있었다[6].

2024년과 2025년도 과제는 전문가 평가에서 최우수 등급을 받았으며, 교육 과정과 아카이브의 정책적 활용 가능성이 긍정적으로 평가되었다.

2026년에는 대면교육을 두 차례 운영하도록 계획하였으며, 상반기 1차 교육은 6월 15일 하루 동안 기존 실습사례를 활용하여 실시하였다. 이론교육에는 14명, 실습훈련에는 역학조사관 등 13명이 참여하였고, 이 중 12명이 만족도 조사에 응답하였다. 응답자의 88% 이상은 교육에 대해 긍정적 평가를 하였으며, 모든 응답자가 추가 교육의 필요성을 제기하였다. 자유 응답에서는 시나리오를 활용한 보고서 작성 훈련의 유용성이 언급되었고, 법적 근거와 교육 목적의 명확화, 그리고 다양한 사례 추가가 개선 요구로 제시되었다. 실습 후에는 교육생이 결과보고서를 작성하고 전문가가 ‘표준 평가 가이드’에 따라 이를 평가하여 조사 보고 역량 향상을 위한 개별 피드백을 제공하였다.

### 4. 사례집 구축과 교육 환류 기반

2025년에는 사례 기반 교육을 지속하기 위한 사례집 구축을 위한 기반도 마련되었다. 연구진은 한국의 산업화 이후 국내에서 발생한 환경보건 관련 건강위해사건 53건을 검토하고, 관련 논문 63건과 보고서 22건을 수집하였다. 입력 항목은 원인미상 건강위해사건의 역학적 특성 입력 양식과 사건별 논문ㆍ보고서 학술정보 입력 양식으로 구분되었다. 원인미상 건강위해사건 14건을 입력 완료하여 사례 표준화의 예시(표 3)를 만들었다[6].

2026년에는 이 사례집을 단순한 자료 목록이 아니라 교육 환류 기반으로 활용하려는 방향이 제시되었다. 사례집에는 역학조사 과정과 결과의 한계, 불확실성 요인, 정책ㆍ제도적 시사점, 전문가 검증 절차가 보완 항목으로 추가되었다. 이는 시나리오 개발, 교육생 보고서 작성, 퍼실리테이터의 피드백, 정규 교육과정 개선을 연결하는 기반으로 기능할 수 있다.

## 논 의

본 과제는 원인미상 비감염성 집단발병을 단순히 새로운 교육 주제가 아니라 정책 사각지대에 대응하기 위한 공중보건 역량의 문제로 재구성했다는 점에서 의미가 있다. 원인 규명 전 단계의 집단발병은 감염병, 환경보건, 화학안전, 재난대응 중 어느 하나의 행정 범주로 즉시 귀속되지 않을 수 있다. 이때 역학조사관에게 필요한 역량은 특정 원인에 대한 전문지식만이 아니라, 불확실한 상황에서 가능한 원인 후보를 열어 두고 조사 우선순위를 정하며, 관계 기관과 자료ㆍ역할을 조정하고, 근거 수준과 불확실성을 명시해 보고하는 능력이다.

이러한 관점은 기후에너지환경부의 환경보건 업무와 질병관리청의 공중보건 조사 업무가 만나는 지점에서 특히 중요하다. 환경보건 업무는 환경오염과 화학물질로 인한 건강영향의 예방ㆍ관리와 연결되지만, 원인미상 집단발병은 원인이 확인되기 전부터 건강영향의 규모와 양상을 파악해야 한다. 따라서 이 교육프로그램은 환경보건 조사를 대체하거나 관할을 주장하기 위한 교육이 아니라, 관할이 확정되기 전의 공백을 줄이고 부처 간 협업을 가능하게 하는 공통 역량 교육으로 자리매김할 필요가 있다.

교육프로그램 개발 측면에서 본 연구의 핵심 산출물은 실제 강좌와 실습 시나리오가 단계적으로 축적되었다는 점이다. 2024년에는 기본 개념과 역학조사 방법을 중심으로 한 온라인 이론교육과 온라인 모의훈련이 마련되었고, 2025년에는 환경독성학, 노출평가, 기후재난 등 실무 적용성이 높은 심화 주제가 추가되었다. 또한 실습 방식은 비대면 모의훈련에서 대면 시나리오 실습으로 발전하였다. 2026년에는 결과보고서 작성과 평가가이드가 추가되어, 단순 참여형 실습을 넘어 조사보고 역량을 평가하고 환류하는 구조로 나아갔다.

특히 사례 기반 시나리오 실습은 이 교육프로그램의 핵심 전략이다. 원인미상 비감염성 집단발병 교육에서는 이미 결론이 난 사건의 결과를 전달하는 것보다, 원인 규명 이전의 불완전한 정보와 상충되는 단서를 경험하게 하는 것이 중요하다. 교육생은 제한된 정보에서 가설을 세우고, 어떤 자료가 더 필요한지 판단하며, 노출평가와 역학자료를 연결하고, 기관 협력과 위험소통을 고려해야 한다. 실습 후 보고서 작성과 피드백은 이러한 판단 과정을 문서화하고 개선하는 기회를 제공한다.

평가 측면에서는 현재 자료가 주로 만족도 조사와 전문가 평가에 머물러 있다는 제한이 있다. 만족도, 이해도, 자기보고식 역량 향상은 교육 수용성과 인식 변화를 보여주지만, 실제 조사역량의 향상이나 현장 행동 변화, 기관 수준의 대응 역량 개선을 직접 입증하지는 않는다. 2026년에 추가된 결과보고서 작성과 평가가이드는 행동 수준 평가로 나아갈 수 있는 중요한 전환점이다. 향후에는 시나리오 보고서 평가, 전후 지식평가, 조사계획 수립 능력, 협업 의사소통, 불확실성 기술 능력 등을 포함한 객관적 평가도구가 필요하다.

본 교육프로그램 개발 과정에서 몇 가지 제한점이 있다. 우선, 만족도 조사의 표본 수가 작고 교육 참여자가 제한적이어서 개발 성과를 일반화하기 어렵다. 또, 평가가 교육생의 만족도와 과제에 대한 전문가 평가로만 이루어져, 교육 전후 지식 변화나 실제 조사역량 향상을 객관적으로 평가한 근거는 없다. 뿐만 아니라 원인미상 건강위해사건의 법적 근거 마련을 통한 조사 권한 명확화, 부처 간 역할 분담, 교육 대상 범위가 아직 충분히 제도화되지 않아서, 교육 내용에서도 이와 관련한 불확실성이 남아 있다.

향후에는 정규 교육 과정화를 위한 표준 교육안, 강사진ㆍ퍼실리테이터 양성체계, 객관적 역량평가 도구, 사례 아카이브 운영체계가 함께 마련되어야 한다. 특히 교육 내용에는 부처 간 협업 훈련이 명시적으로 포함되어야 한다. 예를 들어 교육 시나리오에는 질병관리청, 기후에너지환경부, 지방자치단체, 의료기관, 환경 측정기관, 연구기관이 각각 어떤 정보를 보유하고 어떤 권한과 한계를 갖는지 제시하고, 교육생이 조사 주체, 자료 요청, 현장조사, 위험소통, 보고체계를 설계하도록 할 수 있다. 이는 교육프로그램을 단순 역학기술 교육에서 정책조정 역량 교육으로 확장하는 핵심 요소이다.

결론적으로, 2024–2026년 원인미상 비감염성 집단발병 역학조사 교육프로그램은 정책 사각지대에 놓일 수 있는 사건에 대응할 수 있는 역량을 함양하기 위한 단계적 교육 과정 개발 사례이다. 이 모델은 향후 원인미상 건강위해 집단발병에 대한 국가 공중보건 대응 역량을 강화하고, 공중보건과 환경보건 사이의 협업 기반을 마련하는 데 활용될 수 있다.

## Figures and Tables

**그림 1:** 교육프로그램 개발 및 환류 체계 원인 규명 전 단계의 행정적·과학적 불확실성을 연구회의와 워크숍, 교육자료 제작, 교육 운영과 평가, 환류 및 정규 교육화로 연결하는 교육프로그램 개발 흐름 제시.

**표 1:** 정책 사각지대의 특성과 교육 필요 역량

정책적 특성	현장 문제	교육 필요 역량	교육 구성 요소
원인 규명 전 관할 불명확	감염병, 환경보건, 화학안전, 재난대응 중 어느 절차를 우선 적용할지 불명확	초기 상황평가, 원인 후보 확장, 조사 주체 조정	원인미상 건강위해사건 개념, 감지와 초기대응 교육
부처 간 업무 경계 중첩	공중보건 조사와 환경보건 조사, 지방자치단체 대응이 동시에 필요	기관 간 의사소통, 자료 공유, 역할 분담	협업 시나리오, 조사계획 수립 실습
과학적 불확실성	노출원, 발생기전, 건강영향의 인과성이 즉시 확인되지 않음	가설 설정, 환경노출평가, 독성학적 판단, 불확실성 기술	환경노출평가·환경독성학 이론, 사례 기반 실습
조사결과의 정책 전환 필요	조사결과가 피해자 지원, 환경관리, 제도개선으로 연결되어야 함	보고서 작성, 근거 수준 표현, 정책 시사점 도출	결과보고서 양식, 평가가이드, 피드백

**표 2:** 개발·운영된 교육프로그램의 구성

	이론교육(각 1시간)a)	시나리오 모의 훈련(각 2–4시간)b)
기본	(원인미상) - 건강위해사건의 이해 - 건강위해사건의 집단발병 사례 - 건강위해사건 발생의 관련 요인 - 집단발병의 감지 방법 - 건강위해사건 역학조사 방법 - 역학조사를 위한 환경노출평가의 이해	- 원인미상 중증 폐질환 사건 - 경북 내륙지역 수은 고노출 집락에 대한 역학조사
심화	- 유해인자의 독성과 건강영향에 대한 이해 - 유해인자와 매체 오염 등 노출평가 방법 Part 1 - 유해인자와 매체 오염 등 노출평가 방법 Part 2 - 기후재난 역학조사 - 미래에 집단발병이 가능한 비감염성 건강위해 발굴과 대비	- A대 동물생명과학대학 폐렴 집단발병 사건 - 메트헤모글로빈혈증(Methemoglobinemia) 사건 - 익산 장점마을 집단 암발병 사건 - 산불지역 주민 건강영향 조사

^a)^교재, 동영상을 통한 온라인 교육, 만족도 평가. ^b)^시나리오 훈련 후 결과보고서 작성 및 피드백, 만족도 평가.

**표 3:** 역학조사 사례집에서 원인미상 건강위해사건별 역학적 특성 입력 변수(7개 영역, 82개 변수)

대분류	중분류	변수명
1. 사건 기본 정보	사건 식별	사건ID (분류 체계에 따른 고유번호), 사건명(국문/영문)
	공간 정보	발생 지역(시도-시군구-읍면동), 발생 장소 유형(주거지/공단/학교/의료기관 등), 지리적 범위(반경/면적)
	시간 정보	첫 증상 발생시점(추정), 집단 발생 인지 시점, 최초 신고(제보) 일시, 언론 최초 보도 일시, 공식 조사 착수 일시, 원인 규명 발표 일시
	최초 인지 경로	최초 인지자(주민/의료기관/언론/공무원/연구자 등), 최초 신고 기관, 사회적 인지 경로(언론단체명, 시민단체명, 정부 발표 등)
2. 역학적 특성	건강영향 규모	건강문제 범주(질병명/증후군명), 영향 인구 수(총 노출 인구), 의심 환자 수, 확진 환자 수, 사망자 수, 입원자 수
	역학 지표	발병률, 유병률, 치명률, 초과사망률(해당 시)
	인구학적 특성	성별 분포, 연령대별 분포, 직업별 분포, 고위험군 특성
	임상 특성	주요 증상 및 징후, 증상 발현까지의 잠복기/유도기간, 임상경과 특이사항
	시간적 분포	유행곡선(epidemic curve) 양상, 계절성/주기성 여부
	공간적 분포	지리적 집중도, 공간적 클러스터 존재 여부
3. 노출특성	오염물질	추정 원인물질, 확인된 원인물질, 원인 확인 방법, 원인 물질 특성(화학적, 물리적, 생물학적 등)
	오염원	오염원 종류(공장/광산/매립지/수원 등), 오염원 위치, 오염원 가동 시작 시점, 오염원 개수/규모
	오염 제공자	1차 책임 기업/기관명, 법적 책임 인정 여부
	노출경로	주요 노출 경로(흡입/경구섭취/피부접촉 등), 노출매체(대기/물/토양/식품 등), 노출환경(실내/실외, 직업적/환경적)
	환경 모니터링	환경 시료 채취 일시, 분석 항목, 환경 기준 초과 여부
4. 조사 및 연구	역학조사	역학조사 수행 기관, 역학조사 착수 일시, 역학조사 기간, 조사 방법, 조사 대상자 수, 주요 조사 결과
5. 대응 및 조치	초기대응	최초 대응 기관/조직, 대응 착수 일시
	원인 규명 전 대응	환경조사 실시 여부, 의료 지원 여부, 오염원 가동 중단 여부, 기타 예방 조치
	원인 규명 후 대응	오염원 제거/차단 조치 여부, 주민 건강검진 여부, 환경 정화 사업 여부
	법적/행정적 조치	행정처분 내역, 보상금 규모 및 지급 현황
	장기관리	추적 관찰 실시 여부, 사후 모니터링 체계 여부
6. 사회경제적 영향	피해규모	직접적 경제 피해 규모, 간접적 경제 피해 규모, 이주민 수
7. 논의 및 시사점	제도적 변화	관련 법령 재·개정 여부, 환경보건 기준 변경 여부
		정책 도입 및 변경 여부
	조사의 한계 및 불확실성	역학조사 과정상의 한계, 결과 해석의 불확실성 요인, 미해결 쟁점/추가 조사 필요
	정책·제도적 시사점	제도적 공백/미비점, 정책적 시사점, 재발 방지 제언
